# Florence Nightingale, O.M.

**Published:** 1910-08-20

**Authors:** 


### FLORENCE NIGHTINGALE, O.M.

### HER LIFE AND WORK.

[Though nurses may claim to have a prior interest in the lamentable news, chronicled on Saturday night last, which told us of the death of Florence Nightingale, the heroine of Scutari and the best-known Englishwoman of her age, medical men and institutional workers share that interest to a large extent. In the circumstances the following particulars of Miss Nightingale's eventful life, extracted from the special memoir which appears in this week's issue of The Nursing Mirror, and which, by courtesy of the Editor of that paper, we are enabled to give, will probably be of interest to our readers.—Ed. The Hospital.]

### Early Years.

"Florence Nightingale was born at Florence, May 12, 1820, and was the daughter of Mr. and Mrs. William Shore Nightingale, of Lea Hurst, Derbyshire, and Embley Park, Hampshire. Their other daughter became the second wife of Sir Harry Verney, of Claydon, in Buckinghamshire.

"Her youth was spent partly abroad and partly in England, under the gentlest and happiest home influences. She was taught entirely at home, and under her father's guidance was given a liberal education, not only in Latin, mathematics, and modern languages, but in other things, taught less by books than by the surroundings of life itself; by travel and the study of people, cities, public movements and institutions, and, more than all, by intellectual and cultured companionship under the home roof. Very early Miss Nightingale showed that her own natural tastes lay in the direction of philanthropic work. At first at home, among their poor neighbours, then further afield, in schools, hospitals, and institutions of various kinds, she began a quiet and systematic study of 'the ameliorative treatment of physical and moral distress.' It led her to inspect institutions in various countries of Europe, and she spent some months at the nursing institutions at Kaiserswerth on the Rhine, then recently founded by the good Pastor Fliedrew. It is difficult to overrate the influence of this German Protestant institution on the history of our nursing profession. A talk with one of its gentle sisterhood—the kindly welcome given to a 'weltliche College' for the sake of a common work—helps one to realise that this influence is still active, merged though it is now in the many great modern 'schools' of the present day. Miss Nightingale—herself a Protestant member of the Church of England—allowed no sectarian prejudices to interfere with the work she had at heart. After leaving Kaiserswerth, she went to Paris to learn in its historic institutions, and with the good sisters of St. Vincent de Paul, much that was impossible in those early days of the Protestant hospital on the Rhine. After her return to London she became interested in the sanatorium for governesses in Harley Street, and for some time she devoted to it her energies and private means."

### The Crimean War.

Then came the Crimean War, with Russell's letters on the state of affairs in the peninsula. "It is almost impossible to realise now the wave of dismay, anguish, and indignation which swept over our country in the summer and autumn of 1854, as the accounts continued to arrive from the seat of war of blunders and mismanagement, of the heroism of our soldiers, the awful sufferings of our sick and wounded men. People looked at one another aghast: 'The Catholic French are better off than we are; they have their Sisters of Charity. Are there no women in Protestant England to go forth? . . .'"

It was at this moment that Mr. Sidney Herbert, the Secretary of State for War, wrote to Miss Nightingale his long and memorable letter (see "Life of Florence Nightingale," by Sarah Tooley, p. 105). "There is but one person in England that I know of who would be capable of organising and superintending such a scheme. . . . My question simply is, Would you listen to the request to go out and supervise the whole thing? You would, of course, have plenary authority over all the nurses, and I think I could secure you the fullest assistance and co-operation from the medical staff, and you would also have an unlimited power of drawing on the Government for whatever you think requisite for the success of your mission. . . I must not conceal from you that upon your decision will depend the ultimate success or failure of the plan. Your own personal qualities, your knowledge and your power of administration, and, among greater things, your rank and position in society, give you advantages in such a work which no other person possesses. If this succeeds, an enormous amount of good will be done now, and to persons deserving everything at our hands, and which will multiply the good to all time. . . ." But Miss Nightingale also had been reading the *Times*, and before Mr. Sidney Herbert's letter reached her she had written offering him her services. The two letters crossed each other. There was no time to lose, and after one short week of incredibly hard work for all concerned Miss Nightingale, with her little band of thirty-eight nurses—Kinglake's "Angel Band"—was actually ready to start for the seat of war.

### Work at Scutari.

The work that was done in the Crimea is familiar to all, and for the details of Miss Nightingale's experiences we must refer readers to the memoir in The Nursing Mirror. What hardships the gallant band of lady workers had to endure have never yet been described in full.

"During those first days of her charge Miss Nightingale stood sometimes twenty consecutive hours superintending personally the giving out of stores and the gradual getting things into shape. She herself, so far as was humanly possible, was with the worst and most terrible cases of wounded and with the dying. It must be remembered that she had not been prepared by years of 'training' in surgical work. Yet 'Her nerve is wonderful,' the surgeons reported; and over the sick and dying, night and day, her 'slight form' was seen bending to administer to each case to the best of her power; while with the dying, 'the more awful to every sense each particular case,' the 'greater her self-forgetfulness in the personal care of it would be till death supervened.' 'Before she come,' said a soldier, 'there was such a cussin' and swearin'; and after that it was as holy as a church.' They called her 'the Lady-in-Chief,' as she made her rounds through those dark barrack-wards. 'She could speak to one and to another, and nod and smile to as many more,' was another soldier's report; 'but she couldn't do it to all, you know; we lay there by hundreds; but we could kiss her shadow as it fell, and lay our heads on the pillow again content.'"

On her return from the war she was the heroine of the day, and many stories are told showing the popularity her quiet self-sacrificing zeal had won. At a banquet given to a number of the more prominent officers who had served in the Crimea, the talk turned on the different heroic exploits of the war. Lord Stratford de Redcliffe suggested that it would be interesting to discover whose name, in the opinion of all those assembled, would be longest remembered in British history in connection with the war. As it was not possible to discuss the merits of officers present, Lord Stratford de Redcliffe proposed that each officer should write on a slip of paper the name of which he thought, and that he himself should receive and read the slips and announce the name which had gained the majority of votes. This was done; and when the slips of paper were unfolded, it was found that each officer present had written the same name—"Florence Nightingale."

### Later Years.

"Her life's work was not ended. There was still much to be done. With wonderful strength of character she resumed her private life of quiet, systematic usefulness. There was important work to complete; reports and written evidence to the Commissioners on the working of the Army Medical Department in the Crimea; and later, when the sanitary condition of the Army in India came also under consideration. Her 'Notes on Nursing,' of which, in successive editions, over 100,000 copies have been sold, appeared in 1858. This book preached at the time a new gospel of hygiene to mothers of families and all women who had 'the charge of the personal health of somebody, whether child or invalid'; and many of its axioms have long ago become our household words. Among Miss Nightingale's other writings may be mentioned her 'Notes on Hospitals,' 1859; 'Observations on the Sanitary State of the Army in India,' 1863; 'Notes on Lying-in Institutions,' 1871; 'Life or Death in India?' 1873; 'A Note of Interrogation' (*Fraser's Magazine*, 1873); 'Health at Home' (in the 'Report of the Training of Rural Health Missioners under the North Bucks Technical Education Committee of the Bucks County Council,' 1891-92); and 'Rural Hygiene,' 1894. But much of her most important literary work was in the form of pamphlets, addresses, and Blue-book evidence; and perhaps the most valuable of all was given privately, as counsel to many institutions who claimed her advice and aid, and to persons in whose work she was interested, and whom she wished to help from her own experience. The great practical work of her life, after her return from the Crimea, was the foundation and administration of the Training School for Nurses, for which purpose she had accepted the nation's testimonial of £50,000.

"The Nightingale Fund Training School for Nurses was attached to St. Thomas's Hospital, London, in June 1860. The School was opened with only fifteen probationer nurses, at the old hospital in Southwark, and moved, two years later, when that was sold to the South Eastern Railway Company, to temporary quarters in a music-hall in Surrey Gardens. Here the work of the hospital was carried on till 1871, when the present St. Thomas's Hospital on the Albert Embankment was ready for occupation, and the number of probationers in the Training School was then increased to meet the requirements of the new hospital."

The later years of Miss Nightingale's life were spent in seclusion, chiefly in her house in South Street, Park Lane; for the most part in her room, and on her couch, for her health was broken. She shrank from publicity and refused all honours. Only the Knight of Grace of St. John of Jerusalem she accepted, and a few years ago the late King conferred upon her the high distinction of a membership of the Order of Merit. She was awarded the honorary Freedom of the City of London in 1908, but owing to ill-health was unable to be present at the Guildhall to receive it in person.

### The Funeral.

We understand that the funeral will take place this (Saturday) afternoon in the churchyard at West Willow, Hampshire, where the bodies of Miss Nightingale's father and mother lie. The Dean and Chapter of Westminster Abbey offered a place of interment in the Abbey, but the terms of Miss Nightingale's will are so explicit with reference to her funeral that her executors felt compelled to decline the offer. The funeral will be of a strictly private character, and the number of persons attending it will be limited.

In order that the public may have an opportunity of paying a last tribute of respect, a memorial service will be held in St. Paul's Cathedral to-day at noon. The arrangements have been undertaken by the War Office, but the function will not be of a military character. Admission will be by ticket, for which individuals and bodies wishing to be represented should apply in writing to the Secretary, War Office Memorial Service, Room 109. A limited number of tickets will be issued for the choir and choir gallery. Application should be made to the Secretary, Chapter House, E.C. The King will be represented at the memorial service, and so will the Army Nursing Association and other bodies.

